# Melaninization Reduces Cryptococcus neoformans Susceptibility to Mechanical Stress

**DOI:** 10.1128/msphere.00591-22

**Published:** 2023-01-05

**Authors:** Ellie Rose Mattoon, Radames J. B. Cordero, Arturo Casadevall

**Affiliations:** a Johns Hopkins University, Krieger School of Arts and Sciences, Baltimore, Maryland, USA; b Department of Molecular Microbiology and Immunology, Johns Hopkins Bloomberg School of Public Health, Baltimore, Maryland, USA; University of Georgia

**Keywords:** *Cryptococcus neoformans*, French press, mechanical stress, melanin, mycology, sonication, stress response

## Abstract

Melanin is a complex pigment that is found in various fungal species and is associated with a multitude of protective functions against environmental stresses. In Cryptococcus neoformans, melanin is synthesized from exogenous substrate and deposited in the cell wall. Although melanin is often cited as a protector against mechanical stress, there is a paucity of direct experimental data supporting this claim. To probe whether melanin enhances cellular strength, we used ultrasonic cavitation and French cell press pressure to stress cryptococcal cells and then measured changes in cellular morphology and fragmentation for melanized and nonmelanized C. neoformans cells. Melanized yeast cells exhibited lower rates of fragmentation and greater cell areas than did nonmelanized yeast cells after sonication or French press passage. When subjected to French press passage, both melanized and nonmelanized cells exhibited responses that were dependent on their culture age. Our results indicate that melanization protects against some of the morphological changes, such as fragmentation and cellular shrinkage, that are initiated by mechanical energy derived from either sonic cavitation or French press passage, thus supporting the notion that this pigment provides mechanical strength for fungal cell walls.

**IMPORTANCE** Melanin was shown in prior microbiological experiments to be associated with protection against environmental stressors, and it has often been cited as being associated with mechanical stress protection. However, there is a lack of direct experimentation to confirm this claim. We examined the responses of melanized and nonmelanized C. neoformans cells to sonication and French press passage, and we report differences in outcomes depending not only on melanization status but also on culture age. Such findings have important implications for the design and interpretation of laboratory experiments involving C. neoformans. In addition, the elucidation of some of the mechanical properties of melanin promotes further research into fungal melanin applications in health care and industry.

## INTRODUCTION

Melanin is a complex multifunctional pigment that is found in the cell wall and extracellular vesicles of many fungal species. This pigment is associated with a multitude of protective functions against stresses such as UV radiation, desiccation, and toxic heavy metals (1). Although melanin has often been cited as a protector against mechanical stress (1), there are scant data to directly support this claim. It is known that melanotic fungi can survive in environments at extremely low and extremely high pressures extraterrestrial conditions and deep-sea hydrothermal vents, respectively (2–4). In addition, melanin contributes to virulence, and fungal pathogens likely undergo compressive stresses when invading host tissues (5, 6). Melanization of Venturia inaequalis appressoria is essential for the fungus to penetrate the leaf cuticle of apple, likely by the fungus’ ability to accumulate a high osmotic pressure (7). The invasive hyphal growth of Wangiella dermatitidis in agar exhibited dependence on melanin synthesis (8), and melanin is associated with higher turgor pressures and increased cell wall rigidity in Gaeumannomyces graminis var. *graminis* hyphopodia (9). In addition, melanized Cryptococcus neoformans cells were more likely to survive orbital flight and microgravity conditions than nonmelanized cells (10). Melanization is associated with a reduction in liposome penetration in C. neoformans, a process that is also consistent with increased rigidity (11). Lastly, melanin has been associated with osmotic stress resistance in black yeast (12), which other studies have linked to properties of the fungal cell wall that allow cells to rapidly expand in hypoosmotic environments and avoid rupture (13).

Sonication is a physical process that fragments cell samples using ultrasonic vibrations, which create cavitating bubbles in liquid that release elastic waves and eddies as they collapse (14). As eddies interact with cells, they create a pressure difference, which can cause the cell wall to disintegrate (14). At lower power settings and shorter durations, sonication is often used to remove cell clumps from a sample, with minimal effects on viability (15). However, Saccharomyces cerevisiae cells near cavitation bubbles experienced a fungicidal effect. Other cells experienced a fungistatic effect that increased with longer durations of sonication (16). Earlier assessments of sonic energy’s effects on Saccharomyces ellipsoideus noted that the majority of cells viewed under the microscope were fragmented (17). Recently, our group used sonication to remove the C. neoformans polysaccharide capsule (18) but did not probe the effects on the cell wall.

The French pressure cell press, which is often referred to as simply the French press, is a device used to disrupt the membrane of cells, often for purposes of protein extraction (19). Unlike sonication stress, cells passed through a French press rupture when being passed through a narrow valve at high pressures, as a result of shear forces (20). Cellular organelles often remain intact during this process (19). Sonication differs from French press disruption in that the latter samples are subjected to a single instance of maximum shear force, while sonicated samples may be subject to multiple mechanical shockwaves (19). Like sonication, French press passage can be used to strip the capsule from C. neoformans cells (18).

While melanized fungal cells are relatively more resistant to chemical and electromagnetic radiation stress, the evidence that melaninization contributes to cellular mechanical strength is indirect (1). Hence, we sought to obtain direct evidence for this widely held supposition. In this study, we investigated the responses of melanized and nonmelanized C. neoformans to sonication and French press stress, with the goal of determining whether outcomes differed between the two groups. Melanized cells were more resistant than nonmelanized cells to ultrasonic cavitation and mechanical shear stresses.

## RESULTS

### Cellular mass decreases postsonication.

**FIG 1:**
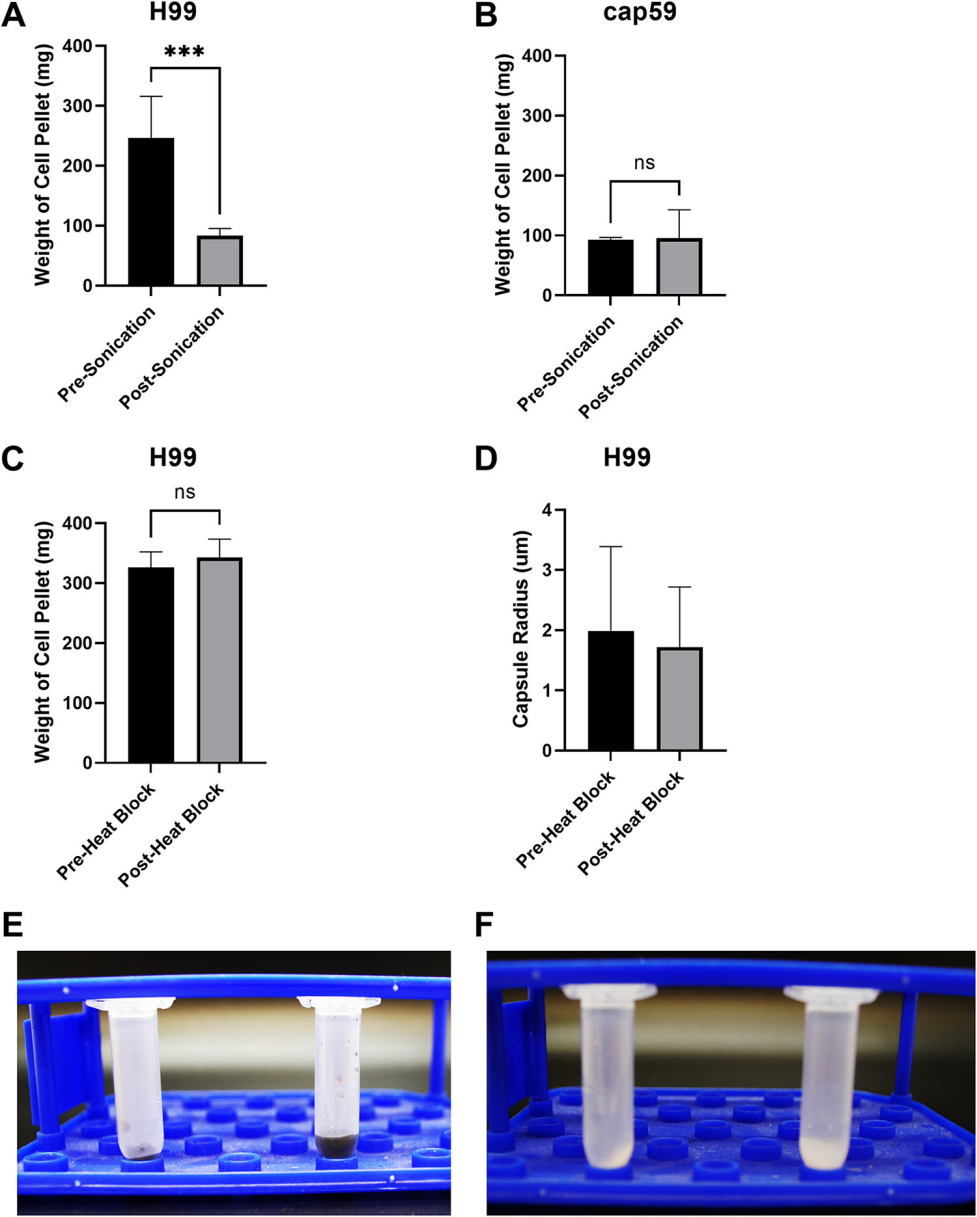
Changes in cellular mass postsonication. (A) Average mass of twice-washed cell pellets of melanized and nonmelanized H99 cells with and without sonication. Cell pellets contained approximately 2 × 10^8^ cells. (B) Average mass of twice-washed cell pellets of melanized and nonmelanized *cap59* mutant cells with and without sonication. Cell pellets contained approximately 2 × 10^8^ cells. (C) Average mass of twice-washed cell pellets of melanized and nonmelanized H99 cells before and after being placed on a heat block at 75°C for 30 min. Cell pellets contained approximately 1.5 × 10^8^ cells. (D) Average capsule radius of melanized and nonmelanized H99 cells before and after being placed on a heat block at 75°C for 30 min. (E) Side-by-side comparison of melanized H99 sonicated (left) and unsonicated (right) cell pellets. (F) Side-by-side comparison of nonmelanized H99 sonicated (left) and unsonicated (right) cell pellets. The brightness and contrast of the images in panels E and F were slightly altered to aid in visualization. Experiments were repeated twice, with comparable results. ***, *P* < 0.001; ns, not significant. All cells were grown in capsule-inducing medium.

**FIG 2:**
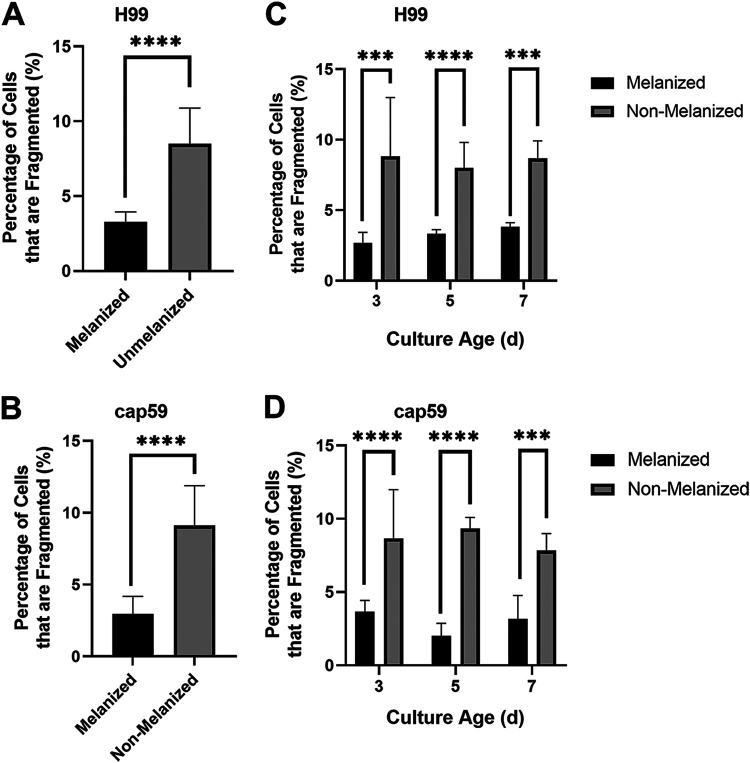
Cell fragmentation following sonication. (A) Percent fragmentation of melanized and nonmelanized H99 cells that underwent three 30-s sonication pulses (*n* = 1,800 for each column) (B) Percent fragmentation of melanized and nonmelanized *cap59* mutant cells that underwent three 30-s sonication pulses (*n* = 1,800 cells for each column) (C) Percent fragmentation of melanized and nonmelanized H99 cells that underwent three 30-s sonication pulses 3 days, 5 days, and 7 days postinoculation (*n* = 600 cells for each column). (D) Percent fragmentation of melanized and nonmelanized *cap59* mutant cells that underwent three 30-s sonication pulses 3 days, 5 days, and 7 days postinoculation (*n* = 600 for each column). Experiments were repeated twice for *cap59* mutant cells and three times for H99 cells, with comparable results. ***, *P* < 0.001; ****, *P* < 0.0001.

Previous researchers have made the observation that the cellular pellets of sonicated samples are visually smaller than the cellular pellets of unsonicated controls, a finding attributed to stripping of the polysaccharide capsule (21). We also observed this phenomenon during experimentation with both melanized and nonmelanized H99 cells, which were grown in a medium that induces capsule production (Fig. 1D and E). To ascertain whether this visual observation was due to a change in volume or mass, treated cells and controls were washed by suspension in minimal medium, followed by centrifugation in microcentrifuge tubes and measurement of the mass of the wet pellet. For both melanized and nonmelanized samples, we observed significant decreases in mass when sonicated and unsonicated samples were compared (Fig. 1A). Given prior findings that the capsule accounts for up to 90% of C. neoformans cell volume and 10% of cell mass (22) and that the process of sonication strips this capsule (18), we repeated the experiment with the acapsular *cap59* mutant. We hypothesized that the mutant would not undergo a change in cell pellet volume, and no significant difference in cellular mass was obtained (Fig. 1B). In addition, the mass of the sonicated H99 culture was similar to the mass of all *cap59* mutant populations. Because these mutants lack capsules, the absence of a mass difference before versus after sonication is consistent with the mass decrease in H99 arising from removal of its cellular capsule. Both sonicated and control H99 and *cap59* mutant samples were examined under a microscope, and sonicated cultures were found to be partially lysed. H99 and *cap59* mutant samples also exhibited similar rates of fragmentation (Fig. 2); therefore, the differences in cell pellet mass cannot be explained by differences in cell fragmentation. The remainder of the sonicated and control cultures were plated; while colonies grew on the control plates, no colonies grew on the sonicated plates (data not shown), consistent with a fungicidal effect from our sonication settings. When this experiment was repeated with a lethal heat shock, there was no significant difference in cellular mass (Fig. 1C) or capsule radius (Fig. 1D), which suggests that cellular death from our sonication treatment was not a major contributor to the loss of the polysaccharide capsule or the decrease in mass.

### Nonmelanized cells are more likely to rupture due to sonication.

**FIG 3:**
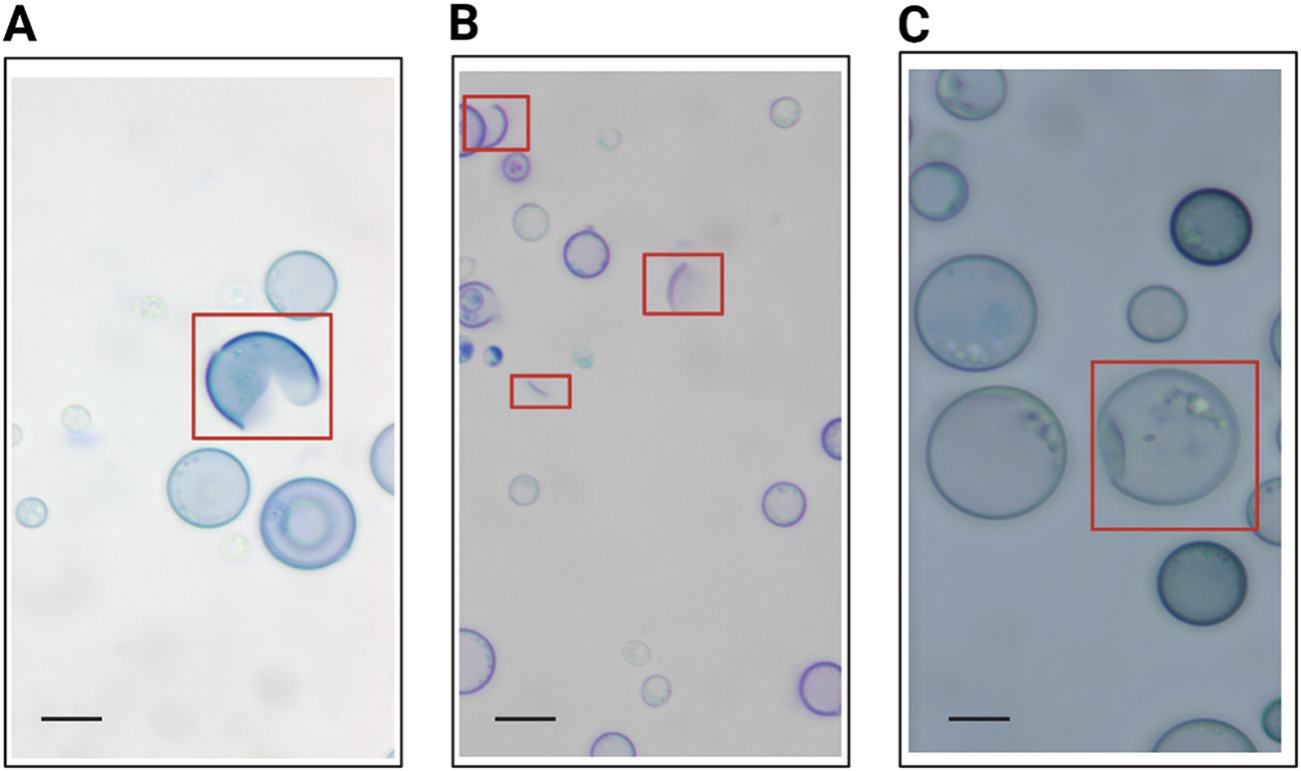
Cellular ruptures defined using bright-field microscopy. (A) “Pacman” cells were mostly intact except for a portion of the cell wall that appeared to be missing. (B) Crescent-shaped fragments appeared as stained arcs with a circumference less than one-half of that of an intact cell. (C) Deformed or “wrinkled pea” cells were identified in both control and treatment cultures and as a result were excluded from analysis. Microscopic images were taken using bright-field microscopy at ×100 magnification, with methylene blue staining. All images shown are of 5-day cell cultures that underwent sonication. Comparable morphologies were identified in cells that underwent French press passage. Scale bars, 6 μm.

**FIG 4:**
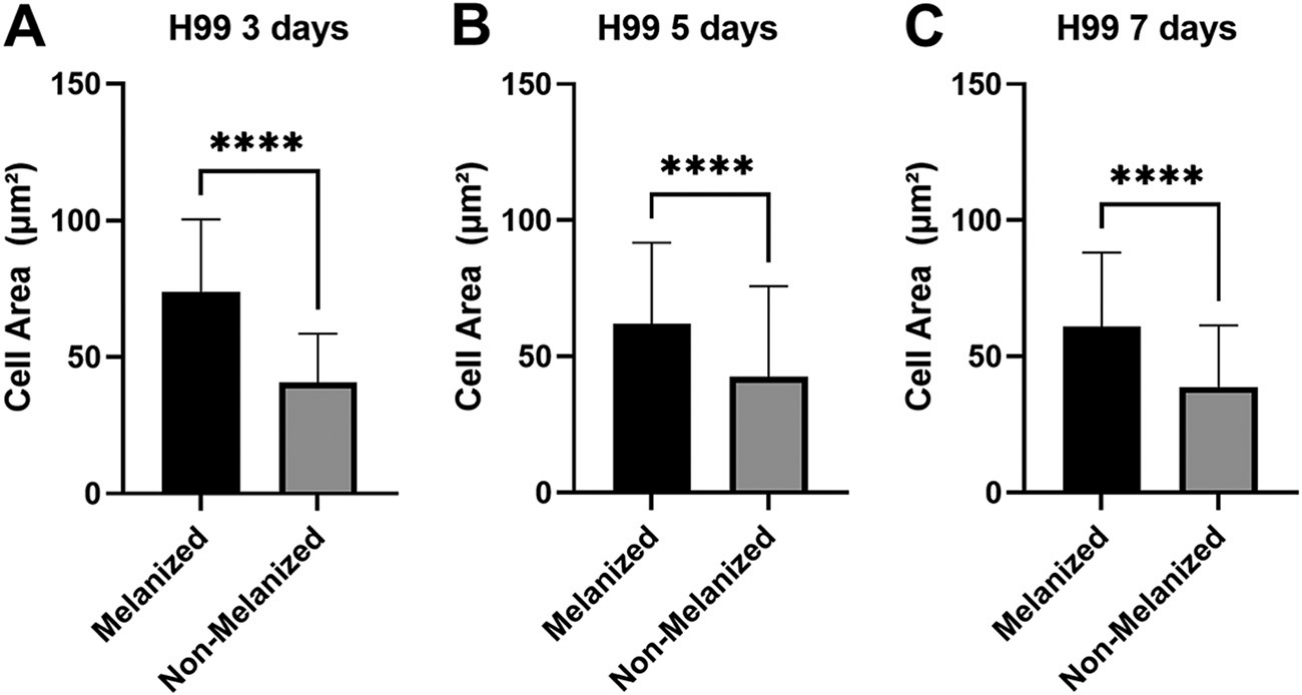
Cell area changes from sonication experiments. (A) Cell areas of both melanized and nonmelanized H99 cells that underwent three 30-s sonication pulses 3 days postinoculation (*n* = 100 cells for each column). (B) Cell areas of both melanized and nonmelanized H99 cells that underwent sonication 5 days postinoculation (*n* = 100 cells for each column). (C) Cell areas of both melanized and nonmelanized H99 cells that underwent sonication 7 days postinoculation (*n* = 100 cells for each column). ****, *P* < 0.0001. This experiment was performed once, with comparable effects for cells that underwent French press passage.

For the purposes of the following experiments, we defined melanized cells as cells grown in medium with l-3,4-dihydroxyphenylalanine (l-DOPA) to induce melanization. Nonmelanized cells were defined as cells grown without l-DOPA. To examine the effects of melanization on cellular susceptibility to fragmentation under sonication stress, melanized and nonmelanized cells that had been separately sonicated were examined by light microscopy. At the specified amplitude, the culture temperature did not rise above 40°C, a temperature that should not affect cell viability (data not shown). Fragmentation was defined and counted in all samples (Fig. 3). For each sample, we counted 200 cells and recorded the proportion of cells that met the criteria for fragmentation (Fig. 3). Next, we studied the effect of sonication on culture age by subjecting cells to sonication 3 days, 5 days, and 7 days postinoculation under either melanization or control (nonmelanization) conditions. When results for all experiments and all culture ages were considered, nonmelanized H99 and *cap59* mutant cells exhibited significantly greater proportions of fragmentation under the microscope than did melanized cells (Fig. 2A and B). To examine the effects of sonication on cell area, bright-field microscopic images of H99 cells stained with methylene blue were analyzed in ImageJ. Magnification and field of view were not adjusted in examinations of cultures of the same age, to ensure comparable area comparisons. Melanized cells exhibited greater cell areas than nonmelanized cells postsonication (Fig. 4A to C). At all culture ages, however, control melanized and nonmelanized cells manifested nonsignificant differences in cell area (*P* = 0.8223, *P* = 0.3518, and *P* = 0.8152 for 3 days, 5 days, and 7 days postinoculation, respectively).

### Nonmelanized cells are more likely to rupture after French press passage.

**FIG 5:**
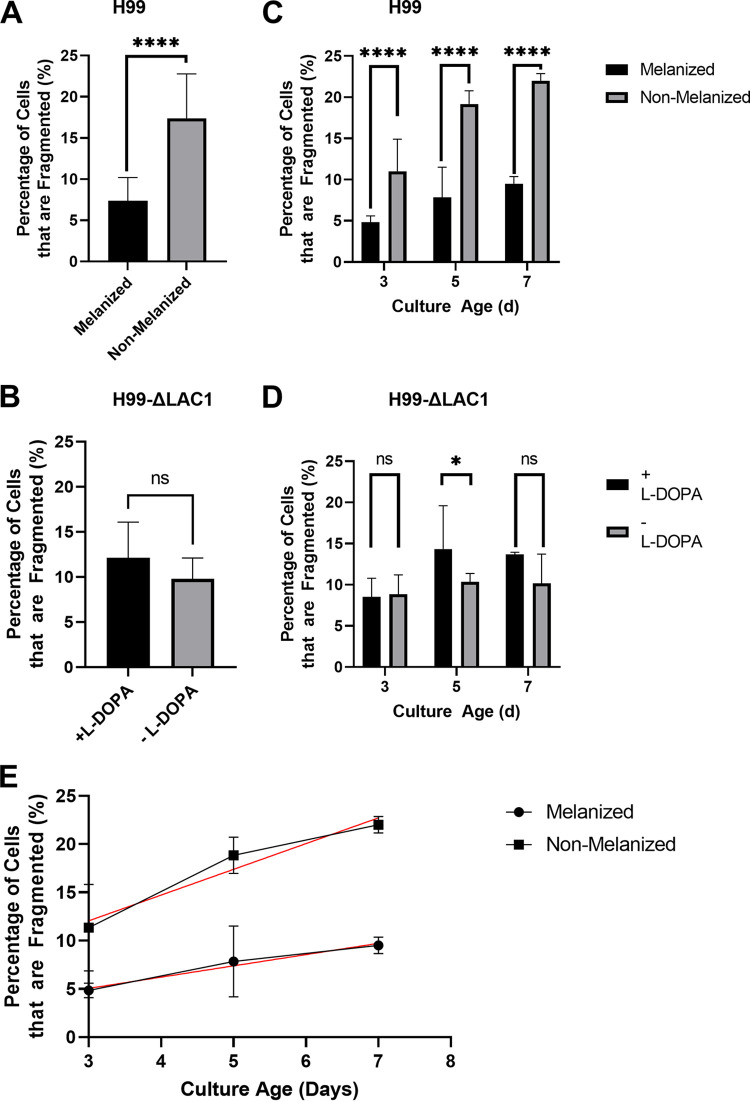
Cell fragmentation following French press passage. (A) Percent fragmentation of melanized and nonmelanized H99 cells that underwent French press passage at 4,964 kPa at all time points (*n* = 1,800 cells for each column). (B) Percent fragmentation of H99-Δ*lac1* mutant cells, grown with or without l-DOPA, that underwent French press passage at 4,964 kPa at all time points (*n* = 1,800 cells for each column). (C) Percent fragmentation of melanized and nonmelanized H99 cells that underwent French press passage at 4,964 kPa 3 days, 5 days, and 7 days postinoculation (*n* = 600 cells for each column). (D) Percent fragmentation of H99-Δ*lac1* mutant cells, grown with or without l-DOPA, that underwent French press passage at 4,964 kPa 3 days, 5 days, and 7 days postinoculation (*n* = 600 cells for each column). Experiments with H99 cells were repeated three times, with comparable results. (E) Percent fragmentation of melanized and nonmelanized H99 cells that underwent French press passage at 4,964 kPa 3 days, 5 days, and 7 days postinoculation (*n* = 600 cells for each data point), regrouped to display trends with respect to culture age. Trends were determined via linear regression (for melanized cultures: slope = 1.167, *R*^2^ = 0.5153; for nonmelanized cultures: slope = 2.667, *R*^2^ = 0.7456). *, *P* < 0.05; ****, *P* < 0.0001; ns, not significant.

**FIG 6:**
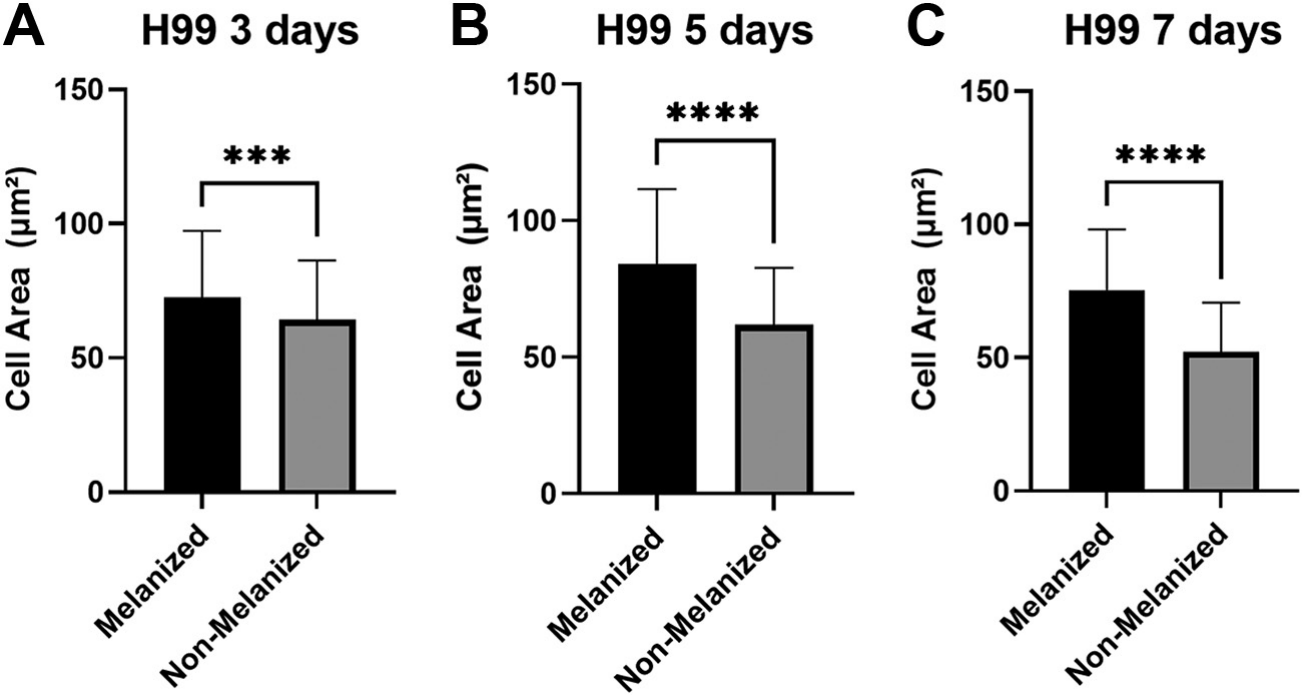
Cell areas after the French press procedure. (A) Cell areas of both melanized and nonmelanized H99 cells that underwent French press passage at 4,964 kPa 3 days postinoculation (*n* = 200 cells for each column). (B) Cell areas of both melanized and nonmelanized H99 cells that underwent French press passage at 4,964 kPa 5 days postinoculation (*n* = 200 cells for each column). (C) Cell areas of both melanized and nonmelanized H99 cells that underwent French press passage at 4,964 kPa 7 days postinoculation (*n* = 200 cells for each column). ***, *P* < 0.001; ****, *P* < 0.0001.

Both melanized and nonmelanized cells passed through a French press were examined under the microscope. For cells of all culture ages, nonmelanized cells exhibited significantly greater proportions of fragmentation under the microscope (Fig. 5A and C). An increase in fragmentation over time was observed in all cultures that had undergone French press stress, regardless of melanization status (Fig. 5E). Melanized cells exhibited greater cell areas than nonmelanized cells after French press passage (Fig. 6A to C); this significance increased between culture ages of 3 days and 5 days (Fig. 6A to C). At all culture ages, however, melanized and nonmelanized cells manifested nonsignificant differences in cell area (*P* = 0.2388, *P* = 0.2152, and *P* = 0.9344 for 3 days, 5 days, and 7 days postinoculation, respectively). For Δ*lac1* mutant cells, which are cells that lack laccase-mediated melanization, there were no significant differences in overall fragmentation (Fig. 5B) except for the 5-day-old cultures, in which cells grown with l-DOPA fragmented more than cells grown without the substrate (Fig. 5D). The Δ*lac1* mutant culture did appear darker in color when grown with l-DOPA; however, because the Δ*lac1* mutant is unable to melanize, this finding was attributed to autopolymerization of l-DOPA itself.

## DISCUSSION

In this study, we examined the widely held assumption that melanization protected fungal cells from mechanical stress using melanized and nonmelanized C. neoformans yeast cells and obtained evidence confirming its veracity. Melanin was shown in prior experiments to have a protective effect against radiative stress (23–25), oxidative stress (26), temperature stress (27–29), and some forms of mechanical stress (8, 9). Determination of melanin’s protective properties during sonication- and French press passage-induced shear stress holds implications for further understanding of melanin’s role in the C. neoformans responses to mechanical stressors in laboratory experiments.

Exposure to ultrasound waves caused changes to the cellular mass, area, and morphology of H99 cells; likewise, French press passage caused changes in H99 cell area and morphology. While changes in cellular mass and/or area appeared to be constant regardless of melanization status, melanized cells were less likely to fragment in response to sonication. While fragmentation is a sufficient cause of cell death from sonication, this is not necessary for cellular death to occur. A prior study found that melanized cells survived less than nonmelanized cells at lower sonication amplitudes (21), which suggests that, while melanin may protect against physical disruption, its protective effects may not apply to cavitation’s fungicidal effects on intact cells.

The principal difference between our control and experimental cells was the presence of l-DOPA, which is a substrate for melanization, in the culture medium. When we repeated our French press experiment with a Δ*lac1* mutant that cannot make melanin, we found nonsignificant differences in fragmentation between cells grown with or without l-DOPA at days 3 and 7 and a borderline increase in fragmentation for cells grown with l-DOPA at day 5. While the cause of the trend in increased fragmentation for laccase-deficient cells grown with l-DOPA at days 5 and 7 is unknown, we noted some darkening of the cultures consistent with autopolymerization and note that this material is different from the pigment synthesized by catalysis in wild-type cells. In any case, this experiment confirms that the increased mechanical stability observed for melanized wild-type cells is not the result of other l-DOPA effects and is consistent with prior observations that l-DOPA is not a nutritionally significant source of carbon or nitrogen (30, 31) and induces few transcriptional changes in C. neoformans cells (32). Because wild-type H99 cells grown in l-DOPA accumulated melanin while control cells did not, our results suggest an association between melanization and increased mechanical strength.

Melanin is not generally recovered from C. neoformans cells until the cultures reach stationary phase after about 3 days of growth, and cells continue to accumulate melanin as late as 8 days postinoculation (28). Our observations that melanization increases its protective effect with culture age following French press passage implies a dose-response relationship consistent with the notion that this phenomenon is correlated with larger quantities of melanin in culture. Sonication can be used to remove the polysaccharide capsule of C. neoformans cells (18), and the supernatants of washed sonicated cultures have been shown to have higher carbohydrate concentrations than those of control cultures (21). These results suggested that most of the mass loss during sonication was from the removal of the capsule. However, we also reported that, regardless of melanization status, C. neoformans cells generally became more susceptible to mechanical stresses with increasing culture age. We hypothesized that cells would become more brittle as they aged, possibly due to the changing composition of the cell wall (33). In addition, these findings are consistent with prior studies indicating that culture age reduces resistance to stress. For example, nonmelanized C. neoformans cells had lower survival rates against UV radiation at greater culture ages (2, 23). One previous study that noted decreasing thermotolerance in older melanized cultures attributed the phenomenon to a decrease in cell wall fluidity due to the continuous deposition of melanin over time (28). Other studies noted that prolonged growth in stationary phase was associated with increased α-1,3-glucan expression in the capsule and changing elasticity in C. neoformans (34). The notion that C. neoformans cells experience changes in their mechanical properties as cultures age has important implications for the design of experiments.

Although one must always be cautious in making mechanistic inferences across large size scales, we note that our microscopic observations that melaninization confers structural strength to melanized cells, relative to nonmelanized cells, are consistent with what we know about the effects of cell wall melanin deposition in C. neoformans. Melanization is known to lead to the formation of covalent bonds between the pigment and cell wall polysaccharide components (35) and chitosan (36), suggesting the formation of cross-linked lattices that could confer increased structural strength to the cell wall. Dopamine-mediated sclerotization of chitin, resulting in the formation of melanin-like material, was associated with a 2.5-fold increase in tensile strength (37). Furthermore, progressive melanization reduces the pore size of the C. neoformans cell wall (38), and the filling of such spaces could provide additional structural strength.

This study is not without limitations. Our analysis focused solely on one pathogenic yeast species and used only two methods of inducing mechanical stress. These methods may not be directly analogous to the mechanical stresses C. neoformans experiences during the invasion of tissue or in the environment. In addition, we studied population effects, and it is possible that the degree of protection against mechanical stress is associated with a single cell’s degree of melanization. However, further research is needed to develop methods that can determine the degree of melanization in a single cell. Nevertheless, the two methods were consistent in that the melanized cells were less vulnerable to fragmentation resulting from shear stress or cavitation.

In summary, melanized cells were less vulnerable to disruption by sonication and French press passage than nonmelanized cells. Melanin is known to be a chemically tough polymer that resists acid degradation, and its presence in the cell wall could provide tensile strength that would reduce the vulnerability of melanized cells to sonication and French press stress, relative to nonmelanized cells. Our results provide direct evidence for the notion that melanin gives cells greater mechanical stability when placed in cell walls.

## MATERIALS AND METHODS

### Species, strains, and media used in this work.

C. neoformans H99, *cap59* mutant, and H99-Δ*lac1* (39) strains were kept frozen as 15% glycerol stocks and precultured on Sabouraud dextrose broth for 48 h prior to inoculation in minimal medium at 30°C. Minimal medium contained 15 mM dextrose, 10 mM MgSO_4_, 29.3 mM KH_2_PO_4_, 13 mM glycine, and 3 mM thiamine-HCl (adjusted to pH 5.5), with or without 1 mM l-DOPA to induce melanization or, in the case of H99-Δ*lac1*, to serve as a control to rule out confounding of our results by the l-DOPA substrate itself.

### Sonication.

Melanized and nonmelanized cultures of either H99 or *cap59* mutant cells were analyzed at 3, 5, and 7 days postinoculation in minimal medium either with or without 1 mM l-DOPA. The yeast cells were counted with a hemocytometer, washed twice with minimal medium, adjusted to 10^8^ cells/mL, and added to glass cuvettes in volumes of 3 mL. Cuvettes were sonicated in a Horn sonicator (sonic dismembrator model 100; Thermo Fisher Scientific, Waltham, MA) at a power of 22 W over an ice bath for three 30-s pulses. This power was determined based on past analyses of the impact of sonication settings on C. neoformans viability and carbohydrate concentrations in samples (21). In addition, a preliminary analysis was performed to determine whether this power produced an optimal amount of fragmentation for further analysis (data not shown). Every sonication experiment conducted featured two control groups; one control group remained in an ice bath for the duration of the sonication process, and the other control group remained at room temperature.

### French press passage.

Melanized and nonmelanized cultures were analyzed at 3, 5, and 7 days postinoculation in minimal medium either with or without 1 mM l-DOPA. The yeast cells were counted with a hemocytometer, washed twice with minimal medium, adjusted to 10^7^ cells/mL, and added to Falcon graduated tubes in volumes of 20 mL. Samples were passed through the French press G-M high-pressure standard cell press (Glen Mills, Inc., Clifton, NJ) once at a setting of 4,964 kPa (720 lb/in^2^). One melanized control and one nonmelanized control were not passed through the French press.

### Cellular changes in mass.

After sonication at an amplitude of 8, 1.0-mL aliquots of treatment and control cultures were washed with 1 mL minimal medium in 2-mL microcentrifuge tubes. The supernatant was removed, and the tubes were weighed on a scale. Five empty microcentrifuge tubes were weighed, and the average of these weights was used to determine the final weight of the cell pellets.

To analyze the effect of heat death on cellular mass, cells were killed on a heat block at 75°C for 35 min. Following heat death, treatment and control cultures were washed with 1 mL minimal medium in 2-mL microcentrifuge tubes. The supernatant was removed, and the tubes were weighed on a scale. Heat-killed cells were also mixed with India ink and imaged with an Olympus AX70 microscope using a Retiga 1300 digital camera and QCapture Suite v2.46 software (QImaging). Capsule measurements were obtained using a quantitative capture analysis program developed by the laboratory (40).

### Analysis of cellular rupture.

After sonication or French press passage, cultures were directly added to slides with methylene blue and imaged with an Olympus AX70 microscope using a Retiga 1300 digital camera and QCapture Suite v2.46 software (QImaging). To visually define cellular fragments, multiple bright-field microscopic images of 5-day cultures were randomly taken at ×100 magnification. To quantify cellular fragments, bright-field microscopic images were taken of all four corners and the center of the slide at ×20 magnification. The number of cellular fragments for 200 cells was then determined visually, starting with the top left corner of the slide and then moving to the top right, bottom right, bottom left, and center if more cells were needed. This process was completed in triplicate for both melanized and nonmelanized cultures, leading to a total of 600 cells counted per treatment condition. Control cultures and, in the case of the sonication experiment, room temperature cultures were examined to confirm the absence of cellular fragments.

### Analysis of cellular area.

Cell area analyses were completed with Fiji software, using methods described previously (41, 42).

### Data analysis.

Data analysis was completed in Prism (GraphPad). The significance of the differences in cell pellet mass was determined using Student's *t* test. The significance of differences in fragmentation between melanized and nonmelanized cells was determined using Fisher’s exact test.
